# Assessing potential impact of gut microbiome disruptions on the environmental stress resilience of indoor-reared *Bombus terrestris*

**DOI:** 10.1371/journal.pone.0290848

**Published:** 2023-11-14

**Authors:** Nazish Roy, Chaerin Kim, Dongmin Lee, Seongeun Yang, Kyeong Yong Lee, Hyung Joo Yoon, Kwang-Sik Lee, Kihyuck Choi

**Affiliations:** 1 Department of Applied Bioscience, Dong-A University, Busan, Republic of Korea; 2 Department of Agricultural Biology, National Academy of Agricultural Science, Wanju, Republic of Korea; University of Illinois Urbana-Champaign, UNITED STATES

## Abstract

Bumblebees are crucial for both natural ecosystems and agriculture, but their decline in distribution and abundance over the past decade is alarming. The global importance of bumblebees in natural ecosystems and agricultural food production cannot be overstated. However, the reported decline over the past decade has led to a surge of interest in understanding and addressing bumblebee population decline. Hence, we aimed to detect disruptions in the gut microbiome of male and worker bumblebees reared indoor and outdoor to assess potential resilience to environmental stress. Using the Illumina MiSeq platform for 16s rRNA amplicon sequencing, we analyzed the gut microbiome of male and worker bees that were raised indoors (designated as the IM and IW group) and those that were raised outdoors (also designated as the OM and OW group). Our results show presence of core bacteria *Neisseriaceae*, *Orbaceae*, *Lactobacillaceae* and *Bifidobacteriaceae* from indoor reared worker bees. However, a higher abundance of *Bifidobacterium* and absence of *Fructobacillus* from indoor reared worker bees was also observed. Indoor-reared male bees had lower diversity and fewer observed OTUs compared to outdoor-reared male bees. Additionally, the relative abundance of Actinobacteriota, Bacteroidota, and Firmicutes was significantly lower in indoor-reared males, while Proteobacteria was significantly increased. Despite this, we did not observe any dysbiosis in the gut microbiota of indoor-reared bumblebees when comparing the role of the gut symbionts among the groups. These results suggest that indoor-reared *Bombus terrestris* may be resilient to environmental stress when used as outdoor pollinators.

## Introduction

Eusocial corbiculate bees are managed valuable resources which provide crucial pollination services and are hence important for food production [1]. The population of these important pollinators has been reported to suffer a global decline [2]. Population decline may be attributed to several factors, such as pathogen spillover from managed pollinators or commercially bred colonies [3–5], toxins and poor nutrition [6]. Research by Hammer et al. (2021) [7], and Kwong and Moran (2016) [1] has shown functional role of bacterial symbionts in bee health. Gut microbiome is of particular interest because of their potential to affect the health and development of the host [8, 9]. The role of gut microbiome has also been elucidated against parasite defense [10, 11]. While the significance of gut microbiota has been acknowledged, the dynamics of its composition remains unclear. Changes in the composition of microbial communities serves as basis of understanding the host development and life processes [12, 13]. Bumblebees are crucial pollinators of wild plants in natural habitats [14], but the structure and composition of gut microbiome is not as well-established as that of honeybees. Hence, focus on examination of bumblebee gut microbiota is required [15]. In bumblebee transmission of symbionts occurs through a single queen [7] but the influence of resource acquisition from different habitats has been reported to change microbial composition affecting health and resistance to pathogens [16]. Parmentier et al. (2018) [17] showed microbial gut community differed between the larvae and adults of wild *Bombus pascuorum*. Variation in the bumblebee gut microbiome community during different stages of development [15, 18–20] and temporal pattern of community stability during old age has been reported [21]. The link between social caste and gut microbiome in *B*. *terrestris* from agriculture landscapes and forest meadows showed that microbial community differed only slightly [22]. The microbial gut community of worker bees from agricultural and semi-natural sites showed little effect of habitat on community composition [10]. Koch and Hemple, 2011 [23] examined microbial flora of worker bumblebee and reported no significant effect of location on the bacterial communities. However, Choi et al. (2023) [24] reported that gut microbiota of indoor reared adult worker bees from two environments differed.

To further investigate the differences in the gut microbiome of bumblebee castes from different habitats, we took advantage of the ability of bumblebees to rear full colonies indoors. This enabled us to compare the gut microbiomes of bees from different habitats. The aim of our study was to determine whether there are differences in the gut microbiomes of indoor and outdoor-reared male and worker bumblebees, and to identify any disruptions in the gut microbiota that may suggest plausible resilience against environmental stress.

## Materials and methods

### Bumblebee sampling and DNA extraction

Bumblebees (*Bombus terrestris*) used in this study were supplied from the Department of Agricultural Biology, National Academy of Agricultural Science, Republic of Korea. The bees were reared in the insectary at 28°C, 65% relative humidity and continuous darkness [25]. Bees raised in insectary were shifted to tomato-planted plastic house 15- days prior to sampling and divided into four groups: indoor-reared male bees (IM), indoor-reared worker bees (IW), outdoor-reared male bees (OM), and outdoor-reared worker bees (OW). Until processed for DNA extraction, samples were stored at -20°C in 75% EtOH. DNA extraction was performed using Wizard genomic DNA purification kit (Promega) by following the manufacturer’s protocol.

### 16s rRNA gene amplification and sequencing

Hypervariable regions V3 & V4 were targeted for 16s rRNA gene amplification using universal primers 341F (5′TCGTCGGCAGCGTCAGATGTGTATAAGAGACAGCCTACGGGNGGCWGCAG-3′) and 805R (5′GTCTCGTGGGCTCGGAGATGTGTATAAGAGACAGGACTACHVGGGTATCTAATCC-3′) with overhang adaptor sequence. Amplification profile used the following conditions: initial denaturation at 95°C for 3 min, followed by 25 cycles of denaturation at 95°C for 30 s, annealing at 55°C for 30 s, and extension at 72°C for 30 s, with a final extension step at 72°C for 5 min. The amplicons were subjected to clean-up by Agencourt AMPure XP PCR Purification system (Beckman Coulter, Brea, USA) for removing PCR primers and primer dimers. The second library preparation and sequencing were performed at NICEM (National Instrumentation Center for Environmental Management), Seoul National University in Seoul, Korea. The libraries were sequenced on Illumina MiSeq platform (Illumina, Inc.). DADA2 (Divisive Amplicon Denoising Algorithm) plug in the QIIME2 (version 2020.08) pipeline was used for low-quality sequence removal, chimeric sequence removal and merging of forward and reverse sequence [26]. Using the de novo VSEARCH algorithm (vsearch cluster-features-de-novo) [27] 97% pairwise nucleotide sequence threshold was used for clustering merged sequences into operational taxonomic units (OTUs) (S1 Fig). The taxonomy of OTUs was assigned using the Naïve Bayes algorithm implemented in the q2-feature-classifier prefitted to the SILVA database (version 138. 1gg_13_5) for the V3-V4 region of 16S rRNA gene [28] (S1 Fig).

### Statistical analyses

Alpha diversity analysis was performed in R using the Shapiro–Wilk normality test, followed by Sudent’s t-test. For beta-diversity analysis PERMANOVA (permutational multivariate analysis of variance) was used with 999 permutations. Pairwise comparisons were done using the Wilcoxon rank-sum test for nonparametric data. For predictive modelling randomforest function in the randomForest R package was used to determine OTUs discriminating the four groups. Differentially abundant taxa were identified by linear discriminant analysis effect size (LEfSE) [29].

## Results

To assess the difference between the gut microbiome of indoor and outdoor reared male and worker bees, we analyzed the bacterial communities from the hind guts of the IM, IW, OM and OW groups (n = 10). A total of 5,022,303 raw reads were generated by amplification of V3-V4 regions. Reads were quality filtered (Q score > 25), denoised and truncated at 290 and 250 bp. After removing chimera and low abundance OTU (20% threshold), 28 OTUs at 97% similarity were obtained (Table 1).

**Table 1:** Sequencing reads and the unique number of OTU after quality control, clustering, and removing chimeras and singleton.

	Domain	Phylum	Class	Order	Family	Genus	Species
OTU1	Bacteria	Proteobacteria	Gammaproteobacteria	Burkholderiales	Neisseriaceae	Unclassified	Unclassified
OTU2	Bacteria	Bacteroidota	Bacteroidia	Flavobacteriales	Weeksellaceae	Apibacter	Apibacter_mensalis
OTU3	Bacteria	Proteobacteria	Gammaproteobacteria	Orbales	Orbaceae	Gilliamella	Unclassified
OTU4	Bacteria	Firmicutes	Bacilli	Lactobacillales	Lactobacillaceae	Lactobacillus	uncultured_Firmicutes
OTU5	Bacteria	Proteobacteria	Alphaproteobacteria	Acetobacterales	Acetobacteraceae	Saccharibacter	Saccharibacter_floricola
OTU6	Bacteria	Proteobacteria	Gammaproteobacteria	Orbales	Orbaceae	Candidatus_Schmidhempelia	Candidatus_Schmidhempelia
OTU7	Bacteria	Actinobacteriota	Actinobacteria	Bifidobacteriales	Bifidobacteriaceae	Bombiscardovia	uncultured_Bifidobacterium
OTU8	Bacteria	Firmicutes	Bacilli	Lactobacillales	Lactobacillaceae	Lactobacillus	Lactobacillus_micheneri
OTU9	Bacteria	Firmicutes	Bacilli	Lactobacillales	Lactobacillaceae	Lactobacillus	Lactobacillus_bombicola
OTU10	Bacteria	Firmicutes	Bacilli	Lactobacillales	Lactobacillaceae	Lactobacillus	Lactobacillus_bombi
OTU11	Bacteria	Firmicutes	Bacilli	Lactobacillales	Lactobacillaceae	Lactobacillus	Unclassified
OTU12	Bacteria	Actinobacteriota	Actinobacteria	Bifidobacteriales	Bifidobacteriaceae	Bifidobacterium	Bifidobacterium_commune
OTU13	Bacteria	Firmicutes	Bacilli	Lactobacillales	Lactobacillaceae	Lactobacillus	Lactobacillus_apis
OTU14	Bacteria	Actinobacteriota	Actinobacteria	Bifidobacteriales	Bifidobacteriaceae	Bifidobacterium	Unclassified
OTU15	Bacteria	Firmicutes	Bacilli	Lactobacillales	Lactobacillaceae	Lactobacillus	Lactobacillus_apinorum
OTU16	Bacteria	Proteobacteria	Gammaproteobacteria	Enterobacterales	Unclassified	Unclassified	Unclassified
OTU17	Bacteria	Firmicutes	Bacilli	Lactobacillales	Vagococcaceae	Vagococcus	Kluyvera_georgiana
OTU18	Bacteria	Firmicutes	Bacilli	Lactobacillales	Enterococcaceae	Enterococcus	Unclassified
OTU19	Bacteria	Actinobacteriota	Actinobacteria	Bifidobacteriales	Bifidobacteriaceae	Bifidobacterium	Unclassified
OTU20	Bacteria	Proteobacteria	Alphaproteobacteria	Sphingomonadales	Sphingomonadaceae	Sphingomonas	Unclassified
OTU21	Bacteria	Proteobacteria	Gammaproteobacteria	Orbales	Orbaceae	Gilliamella	Unclassified
OTU22	Bacteria	Actinobacteriota	Actinobacteria	Bifidobacteriales	Bifidobacteriaceae	Bifidobacterium	Bifidobacterium_bombi
OTU23	Bacteria	Proteobacteria	Alphaproteobacteria	Rhizobiales	Rhizobiaceae	Allorhizobium-Neorhizobium-Pararhizobium-Rhizobium	[Pseudomonas]_geniculata
OTU24	Bacteria	Proteobacteria	Gammaproteobacteria	Enterobacterales	Unclassified	Unclassified	Unclassified
OTU25	Bacteria	Deinococcota	Deinococci	Thermales	Thermaceae	Thermus	Thermus_amyloliquefaciens
OTU26	Bacteria	Firmicutes	Bacilli	Lactobacillales	Leuconostocaceae	Fructobacillus	Unclassified
OTU27	Bacteria	Unclassified	Unclassified	Unclassified	Unclassified	Unclassified	Unclassified
OTU28	Bacteria	Proteobacteria	Gammaproteobacteria	Burkholderiales	Neisseriaceae	Unclassified	Unclassified

### Diversity measures

**Fig 1:**
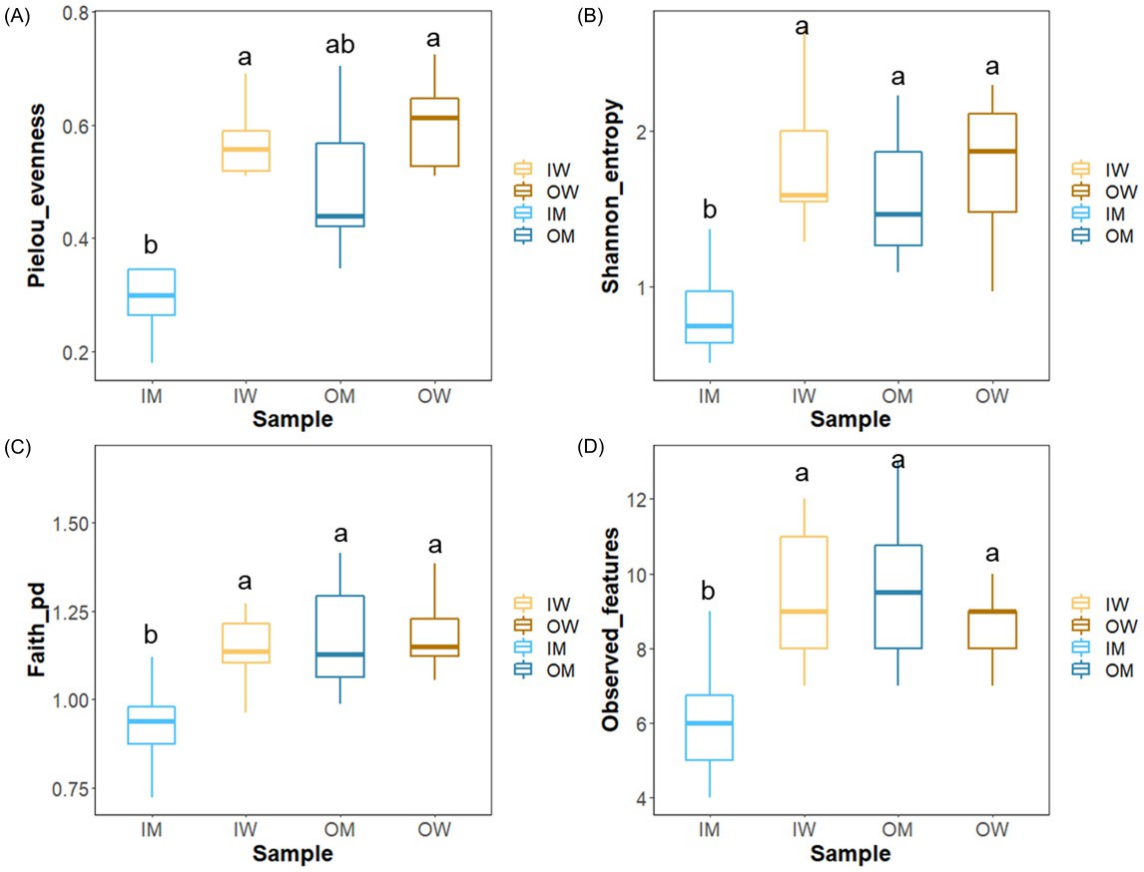
Alpha diversity of *B*. *terrestris* gut microbiome (a) Shannon index (b) Observed OTUs (c) Pielou’s evenness index (d) Faith’s phylogenetic diversity. Different letters indicate significant differences calculated by ANOVA with HSD post hoc test.

**Fig 2:**
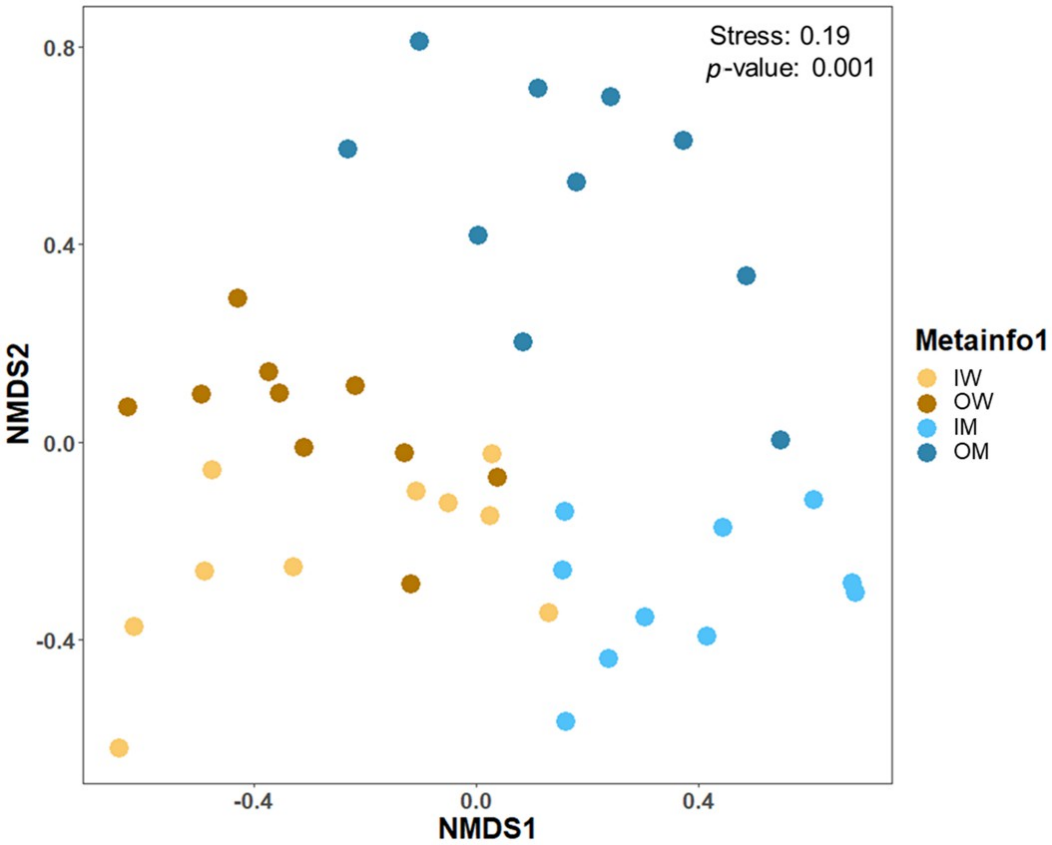
Beta diversity comparison by NMDS, stress = 0.19, based on Bray Curtis dissimilarity showed significant difference among bacterial communities of four groups (PERMANOVA, *p* = 0.001).

We measured alpha diversity indices (Pielou evenness, Shannon entropy, Faith PD and Observed OTUs) of the four groups (Fig 1). The Pielou evenness index value showed significantly low evenness score for IM group compared to IW, OM and OW groups (*p* < 0.001), which showed higher evenness. Similarly, the Shannon entropy values indicated significantly lower diversity in IM in comparison to the IW, OM, and OW groups (*p* < 0.001), which showed higher diversity. The Faith PD values indicated significantly low phylogenetic diversity in IM group in contrast to the IW, OM and OW groups (*p* < 0.001). The observed feature showed significantly lower diversity in IM group compared to the IW, OM and OW groups (*p* < 0.001). Ordination of Bray-Curtis dissimilarity (Fig 2) between gut microbiota of IM, IW, OM and OW groups showed significant difference (PERMANOVA, *p* = 0.001).

### Gut microbiome structure of IM, IW, OM, and OW groups

The gut microbiome analysis of IM, IW, OM, and OW groups revealed a total of 28 OTUs. An overview of the OTUs is presented in Table 2.

**Table 2:** Taxonomic information of the OTUs.

Type	Total Raw Reads	After denoising using DADA2	After denoising, OUT clustring (97%) and removing singleton using DADA2 and VSEARCH
Read	5,022,303	717,330	715,288
OUT		74	28

**Fig 3:**
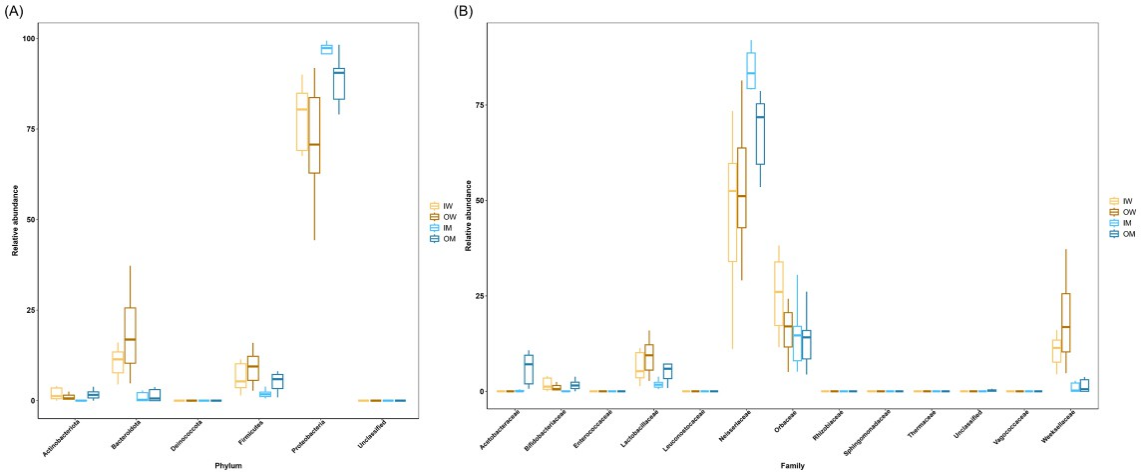
Relative abundance at (A) phylum level and (B) family level of bacterial communities. Different letters indicate significant differences calculated by ANOVA with HSD post hoc test.

**Fig 4:**
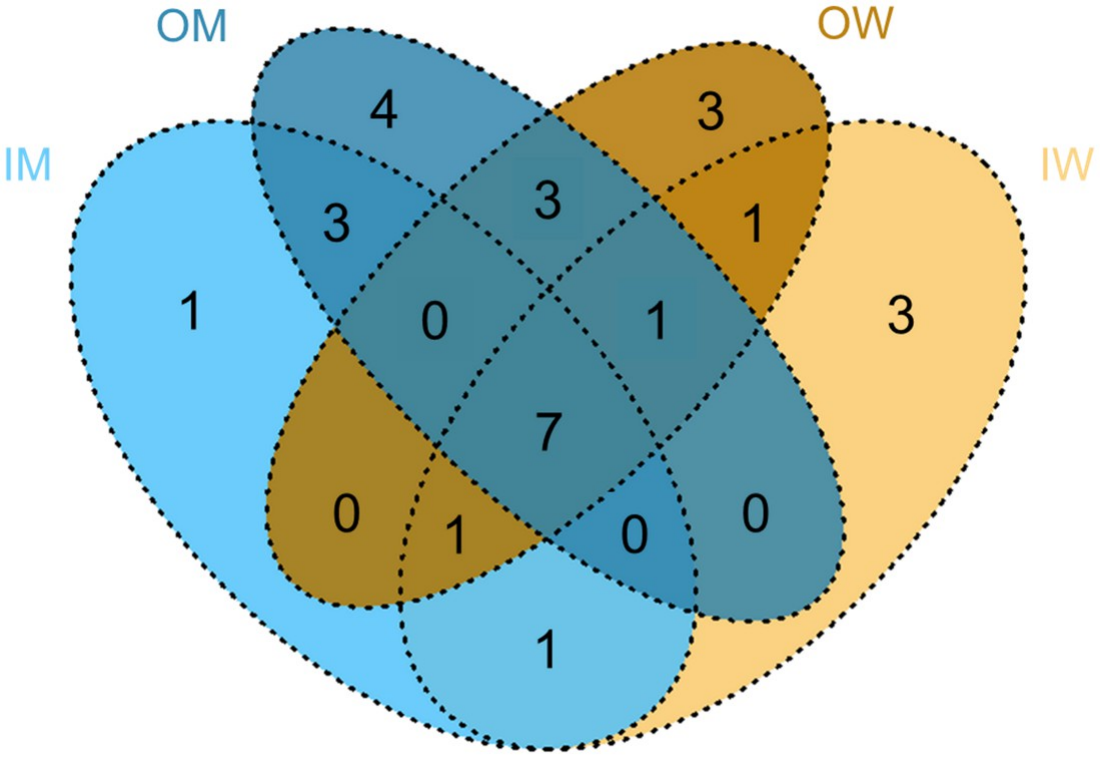
Overlapping OTUs in gut microbiome of bumblebee among four groups displayed by Venn diagram.

The distribution of the six identified phyla (Fig 3A) among the four groups showed that except Deniococcota and the unidentified phyla, the abundance of all other phyla varied among the groups. The exclusive presence and overlap of the OTU observed among the four groups is shown in Fig 4. The abundance of Actinobacteriota, represented by the genera *Bifidobacterium* and *Bombiscardovia*, exhibited a caste-dependent variance in bumblebees reared indoors, but not in those reared outdoor. We did not observe caste or habitat dependence of *Bombiscardovia* or *Bifidobacterium* for gut colonization, except for OTU22 identified as *B*. *bombi* which was exclusively detected in the IM group, and OTU12, *B*. *commune*, which exclusively colonized worker bees irrespective of the habitat. Two OTUs, 19 and 14, of genus *Bifidobacterium* were exclusively detected in IW group. Only one Operational Taxonomic Unit (OTU2) identified as *Apibacter mensalis* represented Bacteroidota and was detected in all samples, indicating an association irrespective of caste and habitat. The abundance of *A*. *mensalis* (OTU2) was significantly higher in worker bees compared to male bees (IW vs IM *p* <0.001, OW vs OM *p* < 0.01) (S1 Fig). Phylum Firmicutes was represented by *Lactobacillus*, *Enterococcus*, and *Fructobacillus*. The relative abundance of Firmicutes was low in the IM group compared to the IW, OM, and OW groups which did not exhibit any significant difference (Fig 3a). Phylum Proteobacteria was the most dominant bacteria detected in all four groups. *Neisseriaceae* (OTU1) and *Orbaceae* (OTU3) were most abundant and present among the four groups (Fig 3B, S1 Fig). The OTU5 identified as *Saccharibacter floricola* from family *Acetobacteraceae* (Table 2) was detected only in male bees from both habitats showing caste dependence irrespective of the habitat (Fig 4). OTU6 identified as *Candidatus_Schmidhempelia* was detected exclusively from outdoor bumblebees irrespective of their habitat (Fig 4).

### RandomForest and LEfSe based identification of caste and habitat associated OTUs

**Fig 5:**
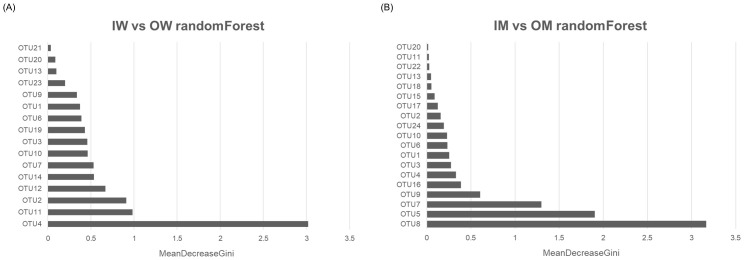
Random Forest analysis of the gut microbiome of (A) IW and OW and (B) IM and OM.

**Fig 6:**
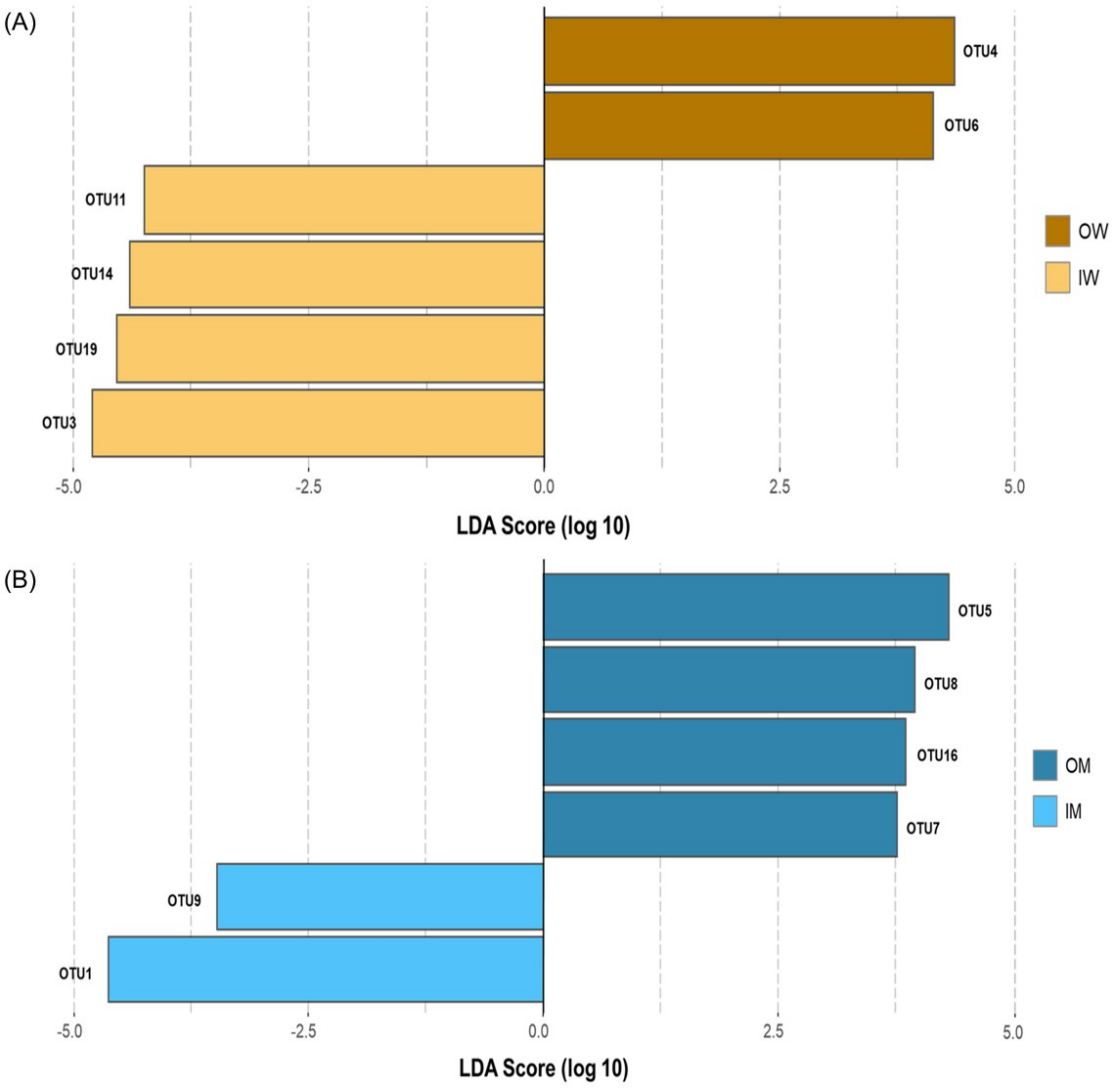
LEfSe identified LDA bar graphs of OTUs differentially abundant between (A) IM and IW groups, (B) OM and OW groups. OTUs statistically significant had (*p*< 0.05) had LDA score (log 10) greater than ±2.5.

**Fig 7:**
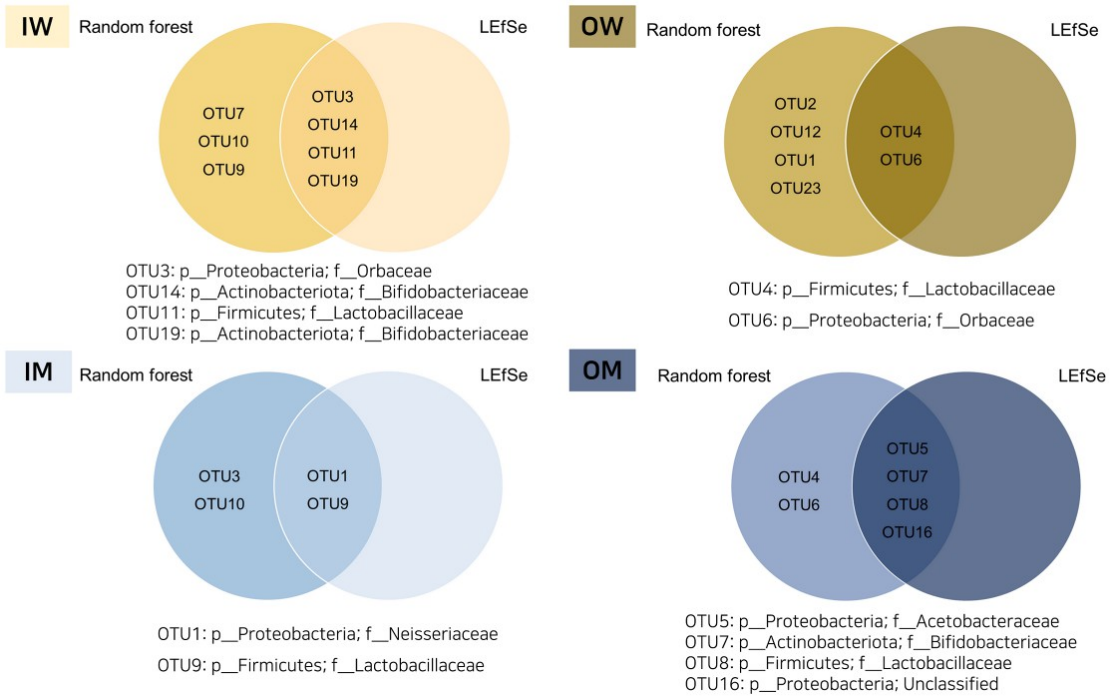
Overlapping OTUs from *in silico* analysis among four groups displayed by Venn diagram.

We used random forest (RF) analysis (Fig 5) and Linear discriminant analysis (Fig 6) to explore the most discriminating OTUs between IW, IM, OW and OM groups. An overlap of OTUs from RF and LEfSe (Fig 7) revealed association of 4 OTUs (3, 14, 11 and 19) with IW group, 2 OTUs (4 and 6) with OW group, 2 OTUs (1 and 9) with IM group and 4 OTUs (6, 7, 8, 16) with OM group. These OTUs were associated with Proteobacteria, Actinobacteriota and Firmicute at phylum level, and *Neisseriaceae*, *Orbaceae*, *Bifidobacteraceae*, *Lactobacillaceae*, *and Acetobacteraceae* at family level.

## Discussion

We document the fitness of gut microbiome from indoor and outdoor reared male and worker bumblebee and infer plausible resilience against environmental stress.

Alpha diversity indexes showed significantly lower diversity in the IM group compared to OM, but we did not observe any significant difference between the OW and IW population (Pielou evenness *p* = 0.999, Shannon entropy *p* = 0.984, Faith PD *p* = 0.961, Observed OUT *p* = 0.704). Significantly lower alpha diversity in male bee was also reported by Krams et al. (2022) [22] across caste in bumblebee. The lower diversity in indoor reared males may have been due to their limited exposure to environmental bacteria but more evidence is required to substantiate this speculation. Beta-diversity analysis showed significant difference in gut flora which is indicative of the higher microbial plasticity associated with the communities harbored by *B*. *terrestris* [23].

Of the three common important operational taxonomic units (OTUs) (7, 14 and 19) identified by both RF and LEfSe (Fig 7) which belonged to Actinobacteriota, OTU7 was detected from all four groups but 2 OTUs (14 and 19) assigned to *Bifidobacteriaceae* (Table 2) were detected only from the IW group (Fig 4). Remarkably, among the five OTUs that were identified as Actinobacteriota, four of them (OTUs 12, 14, 19, and 22) were found to be affiliated with the genus *Bifidobacterium* (Table 2). We observed exclusive association of *Biofidobacterium* with indoor reared bumblebees except *B*. *commune* (OTU 12) which was also detected from OW group (Fig 4). Meeus et al. (2015) [30] noted that core-bacteria of the indoor-reared bumblebees is mainly composed of *Bifidobacterium*. Exclusive detection of *B*. *bombi* (OTU 22) from indoor reared adult worker bumblebee was reported by Choi et al. (2023) [24] who argued that the detection of *Bifidobacteria* was a result of decrease in abundance of core bacteria which allowed better detection based on study by Meeus et al. (2015) [30].

We propose that the gut microbiome of indoor reared bees harboring higher abundance of bifidobacteria compared to the outdoor reared bees may not affect resilience to environmental stresses as bifidobacteria are mainly involved in the carbohydrate metabolism. Zheng et al. (2019) [31] demonstrated in the honeybee gut; genome of *Bifidobacterium* contains repertoires of glycoside hydrolase (GH) genes which were also detected in *Lactobacillus* present. Specifically, their investigation revealed that *B*. *bombi* strain possessed four GH43 genes, while *B*. *commune* did not contain any. Meta-transcriptome of honeybee showed participation of γ-Proteobacteria, Bacilli and Actinobacteria in carbohydrate metabolism [32]. However, there is scarce data which explains the higher abundance of bifidobacteria associated with the indoor reared bees and factors like feed, genetics, and other environmental factors e.g., hygienic conditions may be explored to gain better understanding.

Our results showed that *Fructobacillus* (OTU26) was identified exclusively from OW group (Fig 4). *Fructobacillus* has been reported to dominate gut microbiome of adult worker *B*. *terrestris* from environments with least anthropogenic impact [22]. We propose *Fructobacillus* may have caste dependent colonization irrespective of the habitat. The IW group of our study consumed commercial feed rich in sucrose which could not have sustained *Fructobacillus*. Fructose dependence is attributed to impaired alcohol fermentation in these bacteria, which results from the lack of the *adhE* gene encoding alcohol/acetaldehyde dehydrogenase [33]. Temperature decrease has been reported to increase the fructose concentration in nectar [34] which allows *Fructobacillus* abundance in guts of *B*. *terrestris*. The absence of *Fructobacillus* from IW shows a disruption of gut microbiome which may result in loss of fructose metabolization in cold weather. But we argue that this might only be detrimental to *B*. *terrestris* inhabiting regions with temperatures dropping below freezing point. However, indoor reared *B*. *terrestris* harbor higher abundance of *Bifidobacterium* spp could still thrive and be suitable for survival. Use of *Fructobacillus* as a probiotic prior to onset of freezing temperatures may improve fitness of indoor-reared *B*. *terrestris* by minimizing the disruption of gut microbiome.

Five important OTUs (1, 3, 5, 6 and 16) were identified by RF and LEfSe (Fig 7) which were assigned to Proteobacteria (Table 2). Two (OTUs 1 and 3) of the five were found associated with all four groups.

In our study, *S*. *floricola* (OTU 5) was observed to be exclusively associated with male bees (Fig 4). We observed a significantly high abundance of *Saccharibacter* in OM group compared to IM group (p < 0.01) (S1 Fig). The lower abundance might have been a result of closed breeding system which induced a bottleneck in microbiota of indoor-reared *B*. *terrestris* [30]. Or the high abundance in the OM group may have been a result of immune-mediated apparent competition [35] between a fungal pathogen and *S*. *floricola* which favored growth of *S*. *floricola* in gut of OM group. *S*. *floricola* has been reported to possess type 1 polyketide synthase gene cluster which produces antifungal metabolite responsible for protective effect [36]. The identification of *S*. *floricola* from indoor reared bees implies that the bees possess a degree of adaptation for enduring biotic stress. Studies of bee gut microbiota are limited in their detection of *S*. *floricola* in various bee species. Further research may reveal *S*. *floricola* role in the health and performance of worker bees and other pollinators.

Our results indicated absence of *Candidatus_Schmidhempelia* (OTU6) from indoor-reared bumblebees which may suggest a reduction of the wild microbiota (Fig 4). In comparison to indoor-reared *B*. *terrestris*, Choi et al. (2023) [24] also reported presence of *Candidatus_Schmidhempelia* in adult worker *B*. *terrestris* raised outdoors. We presume that *Candidatus_Schmidhempelia* may rely on a horizontal transmission route, as is the case for most non-core bacteria. *Candidatus_Schmidhempelia* is phylogenetically closely related to *Gilliamella apicola* [37], a core bacterium of gut microbiota of wild and indoor-reared *B*. *terrestris* [30]. The loss of *Candidatus_Schmidhempelia* represents microbiome dysbiosis in indoor-reared bumblebees, which may result in a lack of benefits provided to the host.

Representation of phylum Bacteroidota exclusively by *A*. *mensalis* among all groups was observed in our data (Table 2). Interestingly, our data showed (Table 2) no detection of *Snodgrassella* from any of the four groups. *A*. *mensalis* may utilize the encoded type IX secretion system and gliding motility apparatus to establish biofilms [38]. This mechanism is comparable to that of *S*. *alvi*, which is also an aerobic organism known to create biofilms on the gut wall [39, 40]. The co-occurrence of *Apibacter* alongside *S*. *alvi* has been reported in *Apis cerana*, while *A*. *mellifera* generally does not harbor *S*. *alvi* [41]. Some evidence suggests that there may be competition for a limited ecological niche between *Apibacter* and *S*. *alvi* in bumblebees, as there may be an inverse relationship between their abundance, but the available data on this topic are limited [11, 42, 43]. The co-occurrence and engagement of *S*. *alvi* and *Gilliamella* has been reported due to carboxylate and carbohydrate dependence, respectively [44]. There is limited information available on the metabolic requirements of *A*. *mensalis*, as it is a relatively newly described bacterial species. However, based on the available literature, there is currently no evidence to suggest that *A*. *mensalis* has a carboxylate dependence like *Snodgrassella*. Association of *Apibacter* with *B*. *terrestris* has been reported to decrease infection by *Crithidia bombi*, a trypanosomatid gut parasite [43]. In our study, we found *A*. *mensalis* in both indoor and outdoor bees. As *A*. *mensalis* has been linked to disease suppression, we conclude that the gut symbionts of indoor bees may confer resilience against such biotic stresses.

Although the bumblebee gut communities of the IW, IM, OW and OM groups are different from each other, we did not observe a dysbiosis in indoor reared bumblebees when functions of members of the gut symbionts among the group were compared. We evaluated the comparative fitness potential of the gut communities of indoor-reared bees versus outdoor-reared bees. To achieve this, we focused on the functions of the symbionts provided to the host. However, we acknowledge that our taxonomic classification of symbionts based on partial sequencing of the V3-V4 region had limited resolution. As a result, our assessment of fitness was based on limited knowledge regarding the functional capabilities of bumblebee gut symbionts. Further research is required to investigate the functional roles of various unclassified *Bifidobacterium* spp and *Lactobacillus* spp associated with indoor and outdoor-reared bees with focus on benefits associated with the resilience provided against environmental stress.

## Supporting information

S1 Fig Relative abundance at OUT level of IM, IW, OM, OW groups. Wilcoxon rank-sum test were applied to find significant difference indicated by asterisks (* < 0.05, ** < 0.01, *** < 0.001).
(JPG)

## References

[1] Kwong W.K. and Moran N.A., 2016. Gut microbial communities of social bees. *Nature reviews microbiology*, 14(6), pp. 374–384. doi: 10.1038/nrmicro.2016.43 27140688 PMC5648345

[2] Fürst M.A., Mcmahon D.P., Osborne J.L., Paxton R.J. and Brown M., 2014. Disease associations between honeybees and bumblebees as a threat to wild pollinators. *Nature*, 506(7488), pp. 364–366. doi: 10.1038/nature12977 24553241 PMC3985068

[3] Evison S.E., Roberts K.E., Laurenson L., Pietravalle S., Hui J., Biesmeijer J.C., et al. 2012. Pervasiveness of parasites in pollinators. *PloS one*, 7(1), pp. e30641. doi: 10.1371/journal.pone.0030641 22347356 PMC3273957

[4] Genersch E., Yue C., Fries I. and DE Miranda J.R., 2006. Detection of deformed wing virus, a honeybee viral pathogen, in bumblebees (*Bombus terrestris* and *Bombus pascuorum*) with wing deformities. *Journal of invertebrate pathology*, 91(1), pp. 61–63. 16300785 10.1016/j.jip.2005.10.002

[5] Meeus I., Brown M.J., DE Graaf D.C. and Smagghe G., 2011. Effects of invasive parasites on bumblebee declines. *Conservation Biology*, 25(4), pp. 662–671. doi: 10.1111/j.1523-1739.2011.01707.x 21771075

[6] Vanbergen A.J. and Initiative T.I.P., 2013. Threats to an ecosystem service: pressures on pollinators. *Frontiers in Ecology and the Environment*, 11(5), pp. 251–259.

[7] Hammer T.J., LE E. and Moran N.A., 2021. Thermal niches of specialized gut symbionts: the case of social bees. *Proceedings of the Royal Society B*, 288(1944), pp. 20201480. doi: 10.1098/rspb.2020.1480 33563119 PMC7893241

[8] Engel P. and Moran N.A., 2013. The gut microbiota of insects–diversity in structure and function. *FEMS microbiology reviews*, 37(5), pp. 699–735. doi: 10.1111/1574-6976.12025 23692388

[9] Sommer F. and Bäckhed F., 2013. The gut microbiota—masters of host development and physiology. *Nature reviews microbiology*, 11(4), pp. 227–238. doi: 10.1038/nrmicro2974 23435359

[10] Cariveau D.P., Elijah Powell J., Koch H., Winfree R. and Moran N.A., 2014. Variation in gut microbial communities and its association with pathogen infection in wild bumblebees (*Bombus*). *The ISME journal*, 8(12), pp. 2369–2379. 24763369 10.1038/ismej.2014.68 PMC4260702

[11] Koch H. and Schmid‐Hempel P., 2012. Gut microbiota instead of host genotype drive the specificity in the interaction of a natural host‐parasite system. *Ecology Letters*, 15(10), pp. 1095–1103. doi: 10.1111/j.1461-0248.2012.01831.x 22765311

[12] Koropatnick T.A., Engle J.T., Apicella M.A., Stabb E.V., Goldman W.E., McFall-Ngai M.J., 2004. Microbial factor-mediated development in a host-bacterial mutualism. *Science* 306, pp. 1186–1188. doi: 10.1126/science.1102218 15539604

[13] Hammer T.J., Moran N.A., 2019. Links between metamorphosis and symbiosis in holometabolous insects. *Philosophical Transactions of the Royal Society B*. 374(1783), pp. doi: 10.1098/rstb.2019.0068 . 31438811 PMC6711286

[14] Mänd M., Mänd R. and Williams I.H., 2002. Bumblebees in the agricultural landscape of Estonia. *Agriculture*, *Ecosystems & Environment*, 89(1–2), pp. 69–76.

[15] Su J., Yang X., Lu Q., Liu R., 2021. Antioxidant and anti-tyrosinase activities of bee pollen and identification of active components. *Journal of Apicultural Research*, 60(2), pp. 297–307.

[16] Bosmans L., Pozo M. I., Verreth C., Crauwels S., Wilberts L., Sobhy I. S., et al. 2018. Habitat-specific variation in gut microbial communities and pathogen prevalence in bumblebee queens (*Bombus terrestris*). *PLoS One*, 13(10), pp. e0204612. 30359366 10.1371/journal.pone.0204612 PMC6201867

[17] Parmentier A., Meeus I., VAN Nieuwerburgh F., Deforce D., Vandamme P. and Smagghe G., 2018. A different gut microbial community between larvae and adults of a wild bumblebee nest (*Bombus pascuorum*). *Insect science*, 25(1), pp. 66–74. 27531583 10.1111/1744-7917.12381

[18] Hammer T.J., Le E., Martin A.N. and Moran N.A., 2021. The gut microbiota of bumblebees. *Insectes sociaux*. pp.1–15. doi: 10.1007/s00040-021-00837-1 35342195 PMC8956082

[19] Meeus I., Mommaerts V., Billiet A., Mosallanejad H., Van DE Wiele T., Wäckers F. et al. 2013. Assessment of mutualism between *Bombus terrestris* and its microbiota by use of microcolonies. *Apidologie*, 44, pp. 708–719.

[20] Wang L., Wu J., Li K., Sadd B.M., Guo Y., Zhuang D., et al. 2019. Dynamic changes of gut microbial communities of bumble bee queens through important life stages. *Msystems*, 4(6), pp.e00631–19. doi: 10.1128/mSystems.00631-19 31822600 PMC6906740

[21] Hammer T.J., Easton‐Calabria A. and Moran N.A., 2023. Microbiome assembly and maintenance across the lifespan of bumblebee workers. *Molecular ecology*, 32(3), pp. 724–740. doi: 10.1111/mec.16769 36333950 PMC9871002

[22] Krams R., Gudra D., Popovs S., Willow J., Krama T., Munkevics M., et al. And Contreras Garduño J., 2022. Dominance of Fructose-Associated Fructobacillus in the Gut Microbiome of Bumblebees (*Bombus terrestris*) Inhabiting Natural Forest Meadows. *Insects*, 13(1), pp. 98. 35055941 10.3390/insects13010098 PMC8779478

[23] Koch H. and Schmid-Hempel P., 2011. Bacterial communities in central European bumblebees: low diversity and high specificity. *Microbial Ecology*, 62, pp.121–33. doi: 10.1007/s00248-011-9854-3 21556885

[24] Choi H., Roy N., Kim J., Yoon H.J., Lee K.Y., Lee K. et al. 2023. Dynamics of gut microbiome upon pollination in bumblebee (*Bombus terrestris*). *Journal of Asia-Pacific Entomology*,, pp. 102042.

[25] Yoon H.J., Kim S.E., Lee K.Y., Lee S.B., and Park I.G., 2008. The effect of temperature treatment on the production of worker or queen bumblebees. Journal of Apiculture, 23(4), pp. 283–287.

[26] Bolyen E., Rideout J.R., Dillon M.R., Bokulich N.A., Abnet C.C., Al-Ghalith G.A., et al. 2019. Reproducible, interactive, scalable and extensible microbiome data science using QIIME 2. *Nature biotechnology*, 37(8), pp. 852–857. doi: 10.1038/s41587-019-0209-9 31341288 PMC7015180

[27] Rognes T., Flouri T., Nichols B., Quince C. and Mahé F., 2016. VSEARCH: a versatile open source tool for metagenomics. PeerJ, 4, p. e2584. doi: 10.7717/peerj.2584 27781170 PMC5075697

[28] DeSantis T.Z., Hugenholtz P., Keller K., Brodie E.L., Larsen N., Piceno Y.M., et al. 2006. NAST: a multiple sequence alignment server for comparative analysis of 16S rRNA genes. *Nucleic acids research*, 34(suppl_2), pp. W394–W399. 16845035 10.1093/nar/gkl244 PMC1538769

[29] Segata N., Izard J., Waldron L., Gevers D., Miropolsky L., Garrett W.S. et al. 2011. Metagenomic biomarker discovery and explanation. *Genome biology*, 12, pp. 1–18. doi: 10.1186/gb-2011-12-6-r60 21702898 PMC3218848

[30] Meeus I., Parmentier L., Billiet A., Maebe K., VAN Nieuwerburgh F., Deforce D., et al. 2015. 16S rRNA amplicon sequencing demonstrates that indoor-reared bumblebees (*Bombus terrestris*) harbor a core subset of bacteria normally associated with the wild host. *PloS one*, 10(4), pp. e0125152. 25923917 10.1371/journal.pone.0125152 PMC4414509

[31] Zheng H., Perreau J., Powell J.E., Han B., Zhang Z., Kwong W.K., et al. 2019. Division of labor in honey bee gut microbiota for plant polysaccharide digestion. *Proceedings of the National Academy of Sciences*. 116(51), pp.25909–25916. doi: 10.1073/pnas.1916224116 31776248 PMC6926048

[32] Lee F.J., Rusch D.B., Stewart F.J., Mattila H.R. and Newton I.L., 2015. Saccharide breakdown and fermentation by the honeybee gut microbiome. *Environmental microbiology*, 17(3), pp. 796–815. doi: 10.1111/1462-2920.12526 24905222

[33] Maeno S., Tanizawa Y., Kanesaki Y., Kubota E., Kumar H., Dicks L., et al. 2016. Genomic characterization of a fructophilic bee symbiont *Lactobacillus kunkeei* reveals its niche-specific adaptation. *Systematic and applied microbiology*, 39(8), pp. 516–526. 27776911 10.1016/j.syapm.2016.09.006

[34] Akšić M.F., Tosti T., Nedić N., Marković M., Ličina V., Milojković-Opsenica D. et al. 2015. Influence of frost damage on the sugars and sugar alcohol composition in quince (*Cydonia oblonga* Mill.) floral nectar. *Acta physiologiae plantarum*, 37, pp. 1–11.

[35] Read A.F. and Taylor L.H., 2001. The ecology of genetically diverse infections. *Science*, 292(5519), pp. 1099–1102. doi: 10.1126/science.1059410 11352063

[36] Miller D.L., Smith E.A. and Newton I.L., 2021. A bacterial symbiont protects honeybees from fungal disease. *MBio*, 12(3), pp. 503. 10.1128/mBio.00503-21 PMC8262860 34101488

[37] Martinson V.G., Magoc T., Koch H., SAlzberg S.L. and Moran N.A., 2014. Genomic features of a bumblebee symbiont reflect its host environment. *Applied and Environmental Microbiology*, 80(13), pp. 3793–3803. doi: 10.1128/AEM.00322-14 24747890 PMC4054214

[38] Kita D., Shibata S., Kikuchi Y., Kokubu E., Nakayama K., Saito A. and Ishihara K., 2016. Involvement of the type IX secretion system in *Capnocytophaga ochracea* gliding motility and biofilm formation. *Applied and Environmental Microbiology*, 82(6), pp. 1756–1766. 26729712 10.1128/AEM.03452-15 PMC4784043

[39] Martinson V.G., Moy J. and Moran N.A., 2012. Establishment of characteristic gut bacteria during development of the honeybee worker. *Applied and Environmental Microbiology*, 78(8), pp. 2830–2840. doi: 10.1128/AEM.07810-11 22307297 PMC3318792

[40] Powell J.E., Leonard S.P., Kwong W.K., Engel P. and Moran N.A., 2016. Genome-wide screen identifies host colonization determinants in a bacterial gut symbiont. *Proceedings of the National Academy of Sciences*, 113(48), pp. 13887–13892. 10.1073/pnas.1610856113 PMC5137728 27849596

[41] Kwong W.K., Medina L.A., Koch H., Sing K., Soh E.J.Y., Ascher J.S., et al. 2017. Dynamic microbiome evolution in social bees. *Science advances*, 3(3), pp. e1600513. doi: 10.1126/sciadv.1600513 28435856 PMC5371421

[42] Lim H.C., Chu C., Seufferheld M.J. and Cameron S.A., 2015. Deep sequencing and ecological characterization of gut microbial communities of diverse bumblebee species. *PLoS One*, 10(3), pp. e0118566. doi: 10.1371/journal.pone.0118566 25768110 PMC4359114

[43] Mockler B.K., Kwong W.K., Moran N.A. and Koch H., 2018. Microbiome structure influences infection by the parasite *Crithidia bombi* in bumblebees. *Applied and Environmental Microbiology*, 84(7), pp. 2335. 10.1128/AEM.02335-17 PMC5861814 29374030

[44] Kwong W.K., Engel P., Koch H. and Moran N.A., 2014. Genomics and host specialization of honeybee and bumblebee gut symbionts. *Proceedings of the National Academy of Sciences*, 111(31), pp. 11509–11514. 10.1073/pnas.1405838111 PMC4128107 25053814

